# Linking individual differences in satisfaction with each of Maslow's needs to the Big Five personality traits and Panksepp's primary emotional systems

**DOI:** 10.1016/j.heliyon.2020.e04325

**Published:** 2020-07-23

**Authors:** Christian Montag, Cornelia Sindermann, David Lester, Kenneth L. Davis

**Affiliations:** a Department of Molecular Psychology, Institute of Psychology and Education, Ulm University, Ulm, Germany; b Stockton University, Galloway, NJ 08205-9441, USA; c Pegasus International, Greensboro, NC 27408, USA

**Keywords:** Maslow's hierarchy of needs, Big five of personality, Primary emotional systems, Affective neuroscience theory, Affective neuroscience personality scales, Psychology, Clinical psychology, Personality, Individual differences, Well-being, Neuroscience

## Abstract

Maslow's hierarchy of needs is one of the most impactful theories in motivation psychology and personality science. Therefore, it is surprising that studies linking individual differences in a person's current satisfaction with each of Maslow's needs to the *Big Five personality* traits are rare. In the present study of 850 participants, associations between the *Need Satisfaction Inventory* and the *Big Five* personality traits were examined for the first time. In addition, the administration of the *Affective Neuroscience Personality Scales* provided an evolutionary framework for the present research. Individual differences in the *Need Satisfaction Inventory* were assessed, but participants were also asked about the current importance of each of Maslow's needs in their lives. This latter approach to viewing Maslow's needs (general rated importance of each need in the life of a person) showed strong deviations from Maslow's proposed order in the classic pyramid depicting the hierarchy of needs.

## 1 Introduction

The hierarchy of needs as defined by Maslow (1943, 1970) remains the most visible and widely discussed theories of basic human needs both in and out of psychology (e.g., Benson and Dundis, 2003; Gambrel and Cianci, 2003; Hagerty, 1999; Kiel, 1999; Noltemeyer et al., 2012; Oleson, 2004). Maslow developed his theory as a contribution to the humanistic psychology movement, which was an outcry against the prevailing behavioristic and psychoanalytic mainstream in the first half of the last century (DeCarvalho and Maslow, 1991). Humanistic-oriented psychologists focused on well-being and positive psychology, thereby building a contrast to the then dominating focus on psychopathology.

In his prominent theory on motivation and personality development, Maslow proposed five needs driving human behavior. The most basic of the needs was summed up with the term *Physiological needs* and included, among others, the drives for hunger, thirst and sex. According to Maslow's idea, these physiological needs have to be satisfied before other higher needs in the famous pyramid can be met (see also Table 5, right side). Going through Maslow's classic pyramid from bottom to summit, on the next steps are the S*afety need* and then the *Love and Belongingness* need. The S*afety need* highlights the human need to feel safe and secure, whereas the *Love and Belongingness need* captures the need to have a partner, spouse and/or family, thereby providing company and attachment. At the top of the classic pyramid *Self-esteem* and S*elf-actualization* are located. Whereas S*elf-esteem* can be gained via personal achievements, a prerequisite for the fulfillment of *Self-actualization* is personal growth. Maslow's model is not only a theory of human needs, but also a model of personality development. In particular, the need for S*elf-actualization* can only be met by investing tremendous efforts and after growing up into a mature person. Maslow very much understood that becoming self-actualized meant what philosopher Kierkegaard understood as “to be that self one truly is” (cited after DeCarvalho and Maslow, 1991, p. 41). In other words, *Self-actualization* means also to live up to one's own full potential.

Maslow (1969) modified his five-stage model at a later time by adding *Cognitive* and *Aesthetic needs* as further growth needs below *Self-actualization* but above the step of *Self-Esteem*. Finally, *Transcendence* was put on top of *Self-actualization*, describing a person who ultimately understands the meaning of life (for newer insights in this updated model see Koltko-Rivera (2006)). Kenrick, Griskevicius, Neuberg, and Schaller (2010) revisited Maslow's theory and discussed its relevance and validity against the background of anthropological theory and evolutionary (bio-) psychology. Despite these important additions going beyond Maslow's (early) hierarchy of needs, the present study focuses on his classic five-stage model. This focus is warranted, because the available self-report inventories focus on the original version of Maslow's theory (e.g. Lester, 1990; Taormina and Gao, 2013).

Maslow's stage model can also be seen as an optimal way in which a human develops from early childhood to late adulthood. Whereas first *Physiological* and *Safety needs* play the dominating role in one's own life, in adolescence and adulthood, the growth needs beyond these first steps gain in importance and, at best, fulfillment of *Self-actualization* (or in the later model *Transcendence*) is achieved late in life.

Although Maslow's theory is still an integral part of the present canon of personality psychology, it is surprising that empirical works dealing with Maslow's model are still rather scarce. This might have to do with the relatively limited possibilities to assess individual differences in the satisfaction of Maslow's needs. One option is a self-report questionnaire developed by Lester (1990) assessing individual differences in the satisfaction of each of Maslow's original needs: *Physiological needs, Safety needs, Love and Belongingness need, Self-esteem* and S*elf-actualization*. In this early work by Lester (1990), Maslow's five-stage model was linked to Eysenck's PEN model assessing Psychoticism, Extraversion and Neuroticism. Although the sample size in this early work was small, robust negative correlations between Neuroticism and the current level of satisfaction with all of Maslow's needs was observed, whereas Extraversion was positively associated with the current level of satisfaction in the areas of *Love*, *Self-esteem* and *Self-actualization*. This is also largely in line with another study by Lester et al. (1983) finding similar results (with the exception of no link between Extraversion and *Self-actualization*) using a different measure to assess individual differences in the satisfaction of Maslow's needs, but relying again on Eysenck's PEN model. A study by Saunders et al. (1998) also observed associations between higher negative emotionality (assessed via the Beck Depression/Anxiety and Anger Expression scales) and lower satisfaction with Maslow's needs as measured by Lester's *Need Satisfaction Inventory*. Finally, Taormina and Gao (2013) revisited the neuroticism-need-satisfaction link in context of Maslow's theory and found further support for a robust negative association between higher Neuroticism and lower satisfaction with all of Maslow's needs, although these authors developed their own scale to assess satisfaction with Maslow's needs.

Meanwhile, researchers have applied the *Big Five* personality model to shed light on variables ranging from health behavior to longevity (Bogg and Roberts, 2004; Terracciano et al., 2008). The *Big Five* model has been derived from a lexical approach (for a short historical overview see Montag and Elhai, 2019). In short, statistical analysis of human language led to five broad personality dimensions summarized with the acronym OCEAN: Openness for Experience, Conscientiousness, Extraversion, Agreeableness and Neuroticism. Although the *Big Five* has become somewhat of the gold standard in assessing human personality, it is a purely descriptive model. It cannot explain why humans differ in personality. It simply states that humans differ in these five personality domains.

One prominent biologically-oriented theory shedding light on the fundamental emotional building blocks of the *Big Five* is Panksepp's affective neuroscience theory (Panksepp, 1998).^1^ By applying electrical brain stimulation, endocrinological challenge tests and lesion studies of the mammalian brain (Davis and Montag, 2018), Panksepp carved out seven primary emotional systems believed to provide the foundation of human personality behavior (Davis and Panksepp, 2011). These primary emotional systems are called SEEKING, LUST, CARE and PLAY on the positive side of mammalian emotions, whereas FEAR, ANGER and SADNESS belong to the negative primary emotional systems. In order to not confound these scientifically-defined primary emotional systems with same commonly used terms, they are written in capitals. As all these primary emotions are anchored in ancient subcortical brain regions, they exert their influence on the mammalian (and human) brain in a bottom-up fashion (Davis and Montag, 2019).

All primary emotional systems represent tools for survival, endowing humans (and other mammals) with energy to find life resources such as food and a partner (SEEKING), reproduction (LUST), taking care of the offspring (CARE) and building up social competence and motoric skills (PLAY). Activation of the FEAR system helps mammals to recognize and get out of danger zones and ANGER is activated in cases of defending life resources such as one's own territory or one's own offspring. Finally, the SADNESS system is most strongly active in situations, where mammals experience separation distress. This evolutionary perspective explains well why primary emotional systems have been homologously conserved across species in the mammalian brain.

In an early work by Davis et al. (2003), it was demonstrated that individual differences in primary emotional systems as assessed with the *Affective Neuroscience Personality Scales* (ANPS) could be robustly linked to Big Five personality traits in an USA sample. This study by Davis et al. and a cross-cultural work by Montag and Panksepp (2017), with additional data from Germany and China, demonstrated that SEEKING could be the bottom-up driver of Openness to Experience, PLAY the basis of Extraversion, high CARE/low ANGER the basis of Agreeableness, and high SADNESS/FEAR/ANGER the ancient source driving Neuroticism. Of note, no robust associations between primary emotional systems and Conscientiousness were observed. This was likely because Conscientiousness does not represent an emotion *per se* but rather the capacity to regulate the expression of emotions (Davis and Panksepp, 2018).

Given that Panksepp successfully mapped the neuroanatomy of these primary emotional systems, together with providing a framework for the molecular basis of these primary emotions, Panksepp's affective neuroscience theory can make an important contribution towards answering the question "why" we are the kind of creatures we are. The aforementioned patterns between ANPS and the Big Five traits have been replicated several times (e.g. Giacolini et al., 2017; Montag and Davis, 2018). Moreover, they have not only been investigated with the Big Five traits (Montag et al., 2019), but also investigated in the context of the HEXACO model in Germany and Serbia (Knežević et al., 2020). Therefore, in the present work, these well understood associations between primary emotions and the *Big Five* model of personality will be presented only in the supplementary material (Table S1). The present research will present results linking individual differences in current satisfaction with Maslow's needs both to the *Big Five* model of personality and the underlying primary emotional systems using the ANPS. Bringing these different theoretical perspectives together may help to find a synthesis of the many available theories, each of which aims to shed light on human nature.

With primary emotional action systems seen by Panksepp's affective neuroscience (AN) theory being bottom-up drivers of the *Big Five* traits, it will be interesting to link AN theory to Maslow's hierarchy of needs model. Overlaps between these theories would be interesting because Maslow came up with his theory by studying exceptional, historical persons with great achievements in their lives, whereas Panksepp used a different method by taking the perspective from the inside of the mammalian brain (via deep brain stimulation (DBS), etc.) to carve out mammalian urges arising from the activity of primary emotional systems.

Some of Panksepp's primary emotions and Maslow's needs indeed seem to overlap. Maslow's *Safety need* would mirror evolutionary mechanisms of our brain helping us to stay safe via activation of the FEAR or ANGER circuitry in times of danger. In addition to the wish to be taken CARE of, Maslow's *Need to belong/Love need* might also be anchored in our mammalian nature of feeling and being safer in groups, which explains the evolution of a SADNESS circuit eliciting psychic pain when being alone or losing a beloved person (see links also to depression in Montag et al. (2017) and Panksepp and Watt (2011)). Ultimately, chronic activity of these negative primary emotional systems will hinder reaching high satisfaction levels with Maslow's needs. On the other hand, they are necessary to foster well-being in the long run and are as mentioned above, tools for survival.

To our knowledge, no study so far investigated satisfaction with Maslow's needs in the context of the *Big Five* model. We believe such a study to be relevant, because the *Big Five* model likely belongs among the most important concepts in modern personality psychology, and it is of interest to understand how and if these personality dimensions might impact the likelihood to achieve fulfillment with Maslow's needs.

As reviewed above, only Eysenck's PEN model and the Neuroticism facets out of the *Big Five* model have so far been applied in the context of Maslow's needs, with robust negative associations between all of Maslow's needs and Neuroticism and positive associations between Maslow's *Need to belong/love* and *Self-esteem* and Extraversion. Beyond replication of these associations, the present work is able to establish potential links between satisfaction with Maslow's needs and the *Big Five*'s Openness to Experience, Agreeableness and Conscientiousness dimensions. Given that this has not been studied earlier, the present study is exploratory.

Finally, individual differences in primary emotional systems are linked to current satisfaction levels with Maslow's needs in the present work to provide the present work with an evolutionary framework. Panksepp's AN theory can provide an evolutionary framework because the primary emotional systems of AN theory are homologously conserved in the mammalian brain and anchored in subcortical brain areas (Davis and Montag, 2018). The location of primary emotions in subcortical brain areas suggests an evolutionary basis because the brain architecture might be described in a simplified way by reptilian, mammalian and newer cortical brain layers according to MacLean (1990), with AN theory's primary emotions arising from reptilian and mammalian brain layers (for recent critique of the triune brain concept see Cesario et al., 2020). Given that the primary emotions of SADNESS and FEAR underlie Neuroticism (as well as ANGER to a lesser extent), we expect high SADNESS and FEAR to be negatively linked with satisfaction in Maslow's needs. PLAY has been carved out as the driving force of Extraversion and, therefore, should be positively linked to satisfaction with Maslow's needs. Although the present study is correlational, the personality dimensions (*Big Five* traits and ANPS emotional traits) theoretically should impact on states such as being satisfied (here assessed with Maslow's needs). Therefore, one might see different constellations of personality traits as enabling a person more or less to satisfy Maslow's needs.

In sum, given the surprising lack in the literature investigating Maslow's needs in the context of personality, the present work aims to shed light on how the *Big Five* traits and individual differences in primary emotional systems map onto Maslow's needs. The *Big Five* model was chosen here as it is widely applied in psychological research, and many psychologists would agree that it represents a gold standard. In addition, the ANPS was chosen as a further personality assessment tool because it is rooted in evolutionary psychology and affective neuroscience, an area likely to give new insights into Maslow's theory as discussed also in the works by Kenrick et al. (2010).

## 2 Materials and methods

### 2.1 Sample

A total of 943 people participated in the present online study. In exchange for filling out questionnaires, this online platform provides participants with feedback on their digital consumption, such as smartphone use, and investigates different questions falling in the realm of personality psychology, but also political science. The online platform was promoted via diverse media channels. Of the original 943 participants recruited, 16 were excluded due to implausible specifications about their age (e.g. age of 6 years or younger, or age of above 800 years). Additionally, 6 participants were excluded because they were below 12 years old, and participations was specifically allowed only from the age of 12 years (given permission of the parents/legal guardians). Another 70 participants were excluded because they were not able to follow the instructions correctly. Lastly, one participant was excluded because he/she chose the same answer option throughout one complete self-report measure. A final sample size of 850 participants (210 males, 640 females) remained for the final analyses. *A-priori* power analyses based on Lester's work (1990), in which significant correlations between |.39| and |.62| were found between Extraversion, Neuroticism and the *Need Satisfaction Inventory*, revealed that a sample of at least 79 participants would be necessary to detect such effects (two-tailed, alpha = .05, Power = .95). However, we aimed at investigating a heterogeneous sample and, therefore, recruited more participants in order to be able to draw valid conclusions. The mean age of this sample was 37.04 years (*SD* = 14.59 years), and ages ranged from 12 to 79 years with a median of 36 years. All participants provided electronic informed consent prior to participation. The study was approved by the local ethics committee of Ulm University, Ulm, Germany.

### 2.2 Self-report measures

#### 2.2.1 Need Satisfaction Inventory

For the present study, the 50-item *Need Satisfaction Inventory* (NSI) originally published by Lester (1990) was used in German language. This self-report measure assesses the degree of satisfaction with the five basic need categories according to Maslow's hierarchy of needs. For the translation of the original English version into German language, the items were translated into German and back-translated by two scientists. Afterwards the back-translation and the original version were checked for compatibility. If necessary, adjustments in the German items were implemented after discussion in the group. The final version of the German NSI is presented in the Appendix. The NSI consists of 5 scales, one for each basic need category. These scales are labeled *Physiological*, *Safety* *and* *Security*, *Belonging*, *Esteem*, and *Self-actualization*. For each of the scales, participants are asked to rate their satisfaction with 10 related needs on a 6-point Likert-scale from (-3) = *strongly disagree* to (+3) = *strongly agree* (excluding the (0)). To calculate the respective scores for each scale, the answer options were transformed into a scale ranging from 1 to 6. The internal consistencies (using Cronbach's alpha) fell between .63 (*Physiological needs*) and .82 (*Esteem*) in the present sample. A confirmatory factor analysis (using the maximum likelihood estimator) yielded a CFI (Comparative Fit Index) of .69, a TLI (Tucker Lewis Index) of .68, an RMSEA (Root Mean Square Error of Approximation) of .07, and an SRMR (Standardized Root Mean Square Residual) of .06.

Additionally, participants were asked to rank the importance of the satisfaction of each of the basic need categories (*Physiological*, *Safety and Security*, *Belonging*, *Esteem*, and *Self-actualization*) for themselves. To clarify the constructs, the dimensions were presented with additional information (when necessary). Therefore, we asked for “Belonging and bonding *with friends/family*” or “Self-actualization*, to grow as a person*” (please see below for original wording of all items). Specifically, each basic need category was assigned a number from (1) = *of lowest importance* to (5) = *of highest importance*, while each number was allowed to be assigned only one time.

Here is the instruction including the items we presented to the participants of our study:

The psychologist Maslow carved out several basic needs, playing an important role in personality development. Please state how important the fulfillment of each basic need is for yourself by ordering the terms accordingly: The lowest importance should be marked with a “1”, whereas the need with the highest importance should be marked with a “5”. Please check that each need is marked with one of the numbers 1, 2, 3, 4 or 5 and make sure that each number is used only once.

How important is the fulfillment of each need for you?

Self-esteem and reputation: _______

Belonging and bonding with friends/family: _______

Self-actualization, to grow as a person: _______

Physiological needs such as thirst and hunger: _______

Security: _______

Please check again, that each number 1–5 only occurs once in your order.

#### 2.2.2 Affective Neuroscience Personality Scales

The German version of the Affective Neuroscience Personality Scales (ANPS; Davis et al., 2003; Reuter et al., 2017) was used to assess the six primary emotional traits SEEKING, FEAR, CARE, ANGER, PLAY, SADNESS. Each of these is captured by 14 items, which are answered on a 4-point Likert-scale from (1) = *strongly disagree* to (4) = *strongly agree*. Of note, the questionnaire also includes a Spirituality scale, a lie scale and several filler items. Hence, the questionnaire consists of 110 items in total. The internal consistencies (using Cronbach's alpha) of the scales assessing the primary emotional traits ranged between .67 (SEEKING) and .89 (FEAR) in the present sample.

#### 2.2.3 Big Five Inventory

To assess the *Big Five* traits, the German version of the *Big Five* Inventory (BFI) was used (Rammstedt and Danner, 2016). The German version comprises 45 items (with one additional item in the Agreeableness scale). However, for better comparability with studies using the original 44-item version, in the present study only these 44-items were used. The BFI includes items to measure Extraversion (8 items), Agreeableness (9 items), Conscientiousness (9 items), Neuroticism (8 items), and Openness (to Experience) (10 items). Each item is rated on a 5-point Likert-scale from (1) = *very inapplicable* to (5) = *very applicable*. The internal consistencies (using Cronbach's alpha) of the scales assessing the *Big Five* traits ranged from .70 (Agreeableness) to .86 (Extraversion and Neuroticism).

#### 2.2.4 Statistical analyses

According to the criteria outlined by Miles and Shevlin (2001), a normal distribution could be assumed for the distributions of all scales under investigation (for the complete and for the male and female sub-samples). Therefore, it was decided to use parametric tests throughout this work. First, associations of age and gender with the scales under investigation were investigated by means of Pearson correlations and t-tests (Welch's t-tests were used when necessary). For reasons of transparency, all data are available as a download accompanying the article. Next, partial Pearson correlations (corrected for age; see significant correlations with age presented below) of the NSI with the ANPS and the BFI were calculated (Pearson correlations between the BFI and the ANPS are presented in the supplementary material, Table S1). Moreover, hierarchical linear regression models were carried out to predict the NSI scales by age and gender (first block), the ANPS scales (second block) and the BFI scales (third block). For the different regression models (including variables of block 1, of block 1 and 2, of block 1, 2, and 3) predicting each of the NSI scales, only the model with the highest *R*^2^ will be presented. Finally, the order of the mean values of the NSI scales, the mean values of the importance ratings of the satisfaction of the basic need categories and the original hierarchy of needs are presented.

## 3 Results

### 3.1 Descriptive statistics and associations with age and gender

Age correlated significantly with *Physiological needs* (*r* = .07, *p* = .037), *Safety and Security* (*r* = .20, *p* < .001), *Esteem* (*r* = .26, *p* < .001), and *Self-actualization* (*r* = .13, *p* < .001) scores on the NSI. Moreover, age correlated significantly with FEAR (*r* = -.23, *p* < .001), CARE (*r* = -.17, *p* < .001), ANGER (*r* = -.15, *p* < .001), PLAY (*r* = -.27, *p* < .001), and SADNESS (*r* = -.15, *p* < .001) of the ANPS. Additionally, age correlated significantly with Conscientiousness (*r* = .20, *p* < .001), Neuroticism (*r* = -.17, *p* < .001), and Openness (*r* = .10, *p* = .004) scores of the BFI. This led to the decision to include age as a covariate in later analyses.

Gender differences were found for the following scales: *Physiological*, *Safety* *and* *Security*, *Belonging*, and *Esteem* of the NSI; FEAR, CARE, ANGER, and SADNESS of the ANPS; and Extraversion, Agreeableness, Conscientiousness, and Neuroticism of the BFI. Descriptive statistics for the total, the male sample and the female sample, alongside statistics on differences between males and females, are presented in Table 1.

**Table 1:** Descriptive statistics and statistics on gender differences in the scales under investigation.

	Total Sample (*N* = 850)	Male Sample (*n* = 210)	Female Sample (*n* = 640)	*t* (848)	*p*	Hedge's *g*
	*M* (*SD*)	*M* (*SD*)	*M* (*SD*)			
NSI						

Physiological needs	4.07 (0.73)	4.25 (0.68)	4.02 (0.74)	4.01	<.001	0.319
Safety and Security	4.33 (0.77)	4.55 (0.67)	4.26 (0.79)	5.22	<.001	0.382
Belonging	4.29 (0.80)	4.19 (0.77)	4.33 (0.80)	-2.22^1^	.026	-0.177
Esteem	4.34 (0.82)	4.49 (0.74)	4.29 (0.83)	3.09	.002	0.245
Self-actualization	4.32 (0.80)	4.33 (0.74)	4.31 (0.82)	0.29	.772	0.023

ANPS						

SEEKING	2.80 (0.32)	2.81 (0.31)	2.80 (0.32)	0.38	.701	0.031
FEAR	2.60 (0.53)	2.39 (0.46)	2.67 (0.53)	-7.20	<.001	-0.535
CARE	2.89 (0.38)	2.71 (0.34)	2.95 (0.37)	-8.38^2^	<.001	-0.667
ANGER	2.57 (0.45)	2.49 (0.44)	2.59 (0.45)	-2.80	.005	-0.222
PLAY	2.80 (0.41)	2.81 (0.39)	2.80 (0.42)	0.52	.604	0.041
SADNESS	2.48 (0.39)	2.29 (0.37)	2.54 (0.38)	-8.21	<.001	-0.653

BFI						

Extraversion	3.40 (0.79)	3.24 (0.76)	3.46 (0.79)	-3.49	<.001	-0.277
Agreeableness	3.53 (0.55)	3.44 (0.55)	3.56 (0.55)	-2.86	.004	-0.227
Conscientiousness	3.66 (0.65)	3.56 (0.61)	3.69 (0.67)	-2.54	.011	-0.202
Neuroticism	2.98 (0.79)	2.69 (0.71)	3.08 (0.80)	-6.68	<.001	-0.502
Openness	3.56 (0.60)	3.58 (0.56)	3.56 (0.61)	0.29^3^	.769	0.023

*Note.*^1^ Welch's t-test: df = 415.12; ^2^ Welch's t-test: df = 403.40; ^3^ Welch's t-test: df = 394.39.

### 3.2 Partial correlations between the NSI and the ANPS

As can be seen in Table 2, the FEAR and SADNESS scores of the ANPS were strongly, and the ANGER score moderately, and negatively correlated with all NSI scores. On the other hand, the SEEKING and PLAY scores were moderately and positively correlated with NSI scores. The CARE scale of the ANPS was moderately and positively correlated with the *Belonging* and *Self-actualization* scores of the NSI, as well as slightly and positively correlated with the *Esteem* score.

**Table 2:** Partial Pearson correlations between the NSI scales and the ANPS.

	Physiological needs	Safety and Security	Belonging	Esteem	Self-actualization
SEEKING	*r*_*p*_ = .28 *p* < .001 [.21, .35]	*r*_*p*_ = .27 *p* < .001 [.19, .34]	*r*_*p*_ = .20 *p* < .001 [.14, .26]	*r*_*p*_ = .37 *p* < .001 [.32, .43]	*r*_*p*_ = .42 *p* < .001 [.35, .48]
FEAR	*r*_*p*_ = -.56 *p* < .001 [-.61, -.50]	*r*_*p*_ = -.66 *p* < .001 [-.70, -.62]	*r*_*p*_ = -.34 *p* < .001 [-.40, -.28]	*r*_*p*_ = -.66 *p* < .001 [-.69, -.62]	*r*_*p*_ = -.45 *p* < .001 [-.51, -.39]
CARE	*r*_*p*_ = .01 *p* = .800 [-.07, .08]	*r*_*p*_ = (-).00 *p* = .907 [-.07, .07]	*r*_*p*_ = .24 *p* < .001 [.18, .31]	*r*_*p*_ = .09 *p* = .008 [.02, .17]	*r*_*p*_ = .21 *p* < .001 [.14, .29]
ANGER	*r*_*p*_ = -.29 *p* < .001 [-.36, -.22]	*r*_*p*_ = -.29 *p* < .001 [-.36, -.22]	*r*_*p*_ = -.18 *p* < .001 [-.25, -.12]	*r*_*p*_ = -.29 *p* < .001 [-.36, -.22]	*r*_*p*_ = -.23 *p* < .001 [-.30, -.16]
PLAY	*r*_*p*_ = .41 *p* < .001 [.34, .47]	*r*_*p*_ = .44 *p* < .001 [.38, .50]	*r*_*p*_ = .43 *p* < .001 [.36, .48]	*r*_*p*_ = .48 *p* < .001 [.42, .53]	*r*_*p*_ = .39 *p* < .001 [.33, .45]
SADNESS	*r*_*p*_ = -.53 *p* < .001 [-.58, -.48]	*r*_*p*_ = -.60 *p* < .001 [-.64, -.55]	*r*_*p*_ = -.33 *p* < .001 [-.39, -.26]	*r*_*p*_ = -.57 *p* < .001 [-.62, -.52]	*r*_*p*_ = -.40 *p* < .001 [-.46, -.33]

*Note.* All correlations are corrected for age. BCs 95% confidence intervals are presented (based on 1000 samples).

### 3.3 Partial correlations between the NSI and the BFI

As can be seen in Table 3, the Neuroticism score of the BFI was negatively correlated with the NSI scores. All other BFI scores were significantly and positively correlated with all NSI scores, except Openness, which was not significantly correlated with the *Physiological*, *Safety and Security* scores. In greater detail, Extraversion was most strongly correlated with the *Esteem* score of the NSI, Agreeableness was most strongly correlated with the *Belonging* score of the NSI, Conscientiousness was most strongly correlated with the *Self-actualization* score of the NSI, and Openness was most strongly correlated with the *Self-actualization* score of the NSI. Lastly, Neuroticism was strongly and negatively correlated with most of the NSI scores, but the strongest association was found for the *Esteem* score.

**Table 3:** Partial Pearson correlations between the NSI scales and the BFI scales.

	Physiological needs	Safety and Security	Belonging	Esteem	Self-actualization
Extraversion	*r*_*p*_ = .29 *p* < .001 [.22, .35]	*r*_*p*_ = .30 *p* < .001 [.24, .36]	*r*_*p*_ = .40 *p* < .001 [.33, .46]	*r*_*p*_ = .52 *p* < .001 [.46, .57]	*r*_*p*_ = .46 *p* < .001 [.40, .51]
Agreeableness	*r*_*p*_ = .20 *p* < .001 [.13, .27]	*r*_*p*_ = .25 *p* < .001 [.17, .32]	*r*_*p*_ = .35 *p* < .001 [.28, .41]	*r*_*p*_ = .27 *p* < .001 [.20, .33]	*r*_*p*_ = .29 *p* < .001 [.22, .36]
Conscientiousness	*r*_*p*_ = .28 *p* < .001 [.21, .34]	*r*_*p*_ = .34 *p* < .001 [.28, .40]	*r*_*p*_ = .24 *p* < .001 [.17, .31]	*r*_*p*_ = .38 *p* < .001 [.32, .44]	*r*_*p*_ = .47 *p* < .001 [.41, .52]
Neuroticism	*r*_*p*_ = -.51 *p* < .001 [-.56, -.45]	*r*_*p*_ = -.57 *p* < .001 [-.62, -.52]	*r*_*p*_ = -.32 *p* < .001 [-.39, -.25]	*r*_*p*_ = -.61 *p* < .001 [-.65, -.57]	*r*_*p*_ = -.44 *p* < .001 [-.50, -.38]
Openness	*r*_*p*_ = .04 *p* = .233 [-.03, .11]	*r*_*p*_ = .03 *p* = .393 [-.04, .11]	*r*_*p*_ = .10 *p* = .005 [.03, .17]	*r*_*p*_ = .14 *p* < .001 [.07, .21]	*r*_*p*_ = .20 *p* < .001 [.13, .27]

*Note.* All correlations are corrected for age. BCs 95% confidence intervals are presented (based on 1000 samples).

Please find correlations on the ANPS and the Big Five in the supplementary material (Table S1).

### 3.4 Hierarchical regression models to predict the NSI scales

For the three regression models predicting the NSI *Physiological* score, the third model (including variables of each of the three blocks) showed the highest *R*^2^ (adjusted *R*^2^ = .398; *F* (13,836) = 44.15, *p* < .001). Also for the three regression models predicting the NSI *Safety and Security* score, the third model (including variables of each of the three blocks) showed the highest *R*^2^ (adjusted R^2^ = .549; *F* (13,836) = 80.54, *p* < .001). The same was true for the regression models predicting the NSI *Belonging* score (adjusted *R*^2^ = .315; *F* (13,836) = 31.03, *p* < .001), *Esteem* score (adjusted *R*^2^ = .621; *F* (13,836) = 108.21, *p* < .001), and *Self-actualization* score (*R*^2^ = .483; *F* (13,836) = 62.08, *p* < .001). The regression models for each NSI scale, including all variables of each block (age and gender, ANPS scales, BFI scales), are presented in Table 4.

**Table 4:** Regression models predicting the NSI scales by age, gender, the ANPS, and the BFI scales.

	Physiological needs			Safety and Security			Belonging			Esteem			Self-actualization		
	β	*T*	*p*	β	*T*	*p*	β	*T*	*p*	β	*T*	*p*	β	*T*	*p*
Intercept		11.90	<.001		13.21	<.001		4.25	<.001		10.58	<.001		2.87	.004
Age	-.007	-0.22	.826	.082	3.07	.002	.021	0.63	.529	.128	5.22	<.001	.033	1.17	.242
Gender	-.022	-0.74	.461	-.026	-1.00	.315	.058	1.85	.065	-.015	-0.63	.530	-.011	-0.40	.687
SEEKING	.097	2.86	.004	.046	1.56	.119	-.046	-1.28	.200	.081	3.02	.003	.166	5.30	<.001
FEAR	-.239	-4.98	<.001	-.393	-9.46	<.001	-.055	-1.07	.284	-.266	-6.98	<.001	-.121	-2.73	.006
CARE	-.002	-0.05	.960	-.020	-0.71	.481	.107	3.03	.003	.031	1.18	.237	.106	3.45	<.001
ANGER	-.032	-0.91	.365	.062	1.99	.047	.050	1.32	.189	.027	0.94	.349	.035	1.06	.289
PLAY	.158	4.28	<.001	.163	5.08	<.001	.180	4.56	<.001	.056	1.92	.055	.019	0.56	.577
SADNESS	-.179	-4.11	<.001	-.170	-4.51	<.001	-.192	-4.14	<.001	-.131	-3.79	<.001	-.109	-2.71	.007
Extraversion	-.011	-0.31	.759	-.059	-1.99	.047	.174	4.73	<.001	.201	7.35	<.001	.159	4.99	<.001
Agreeableness	.008	0.23	.821	.085	2.77	.006	.193	5.11	<.001	.031	1.09	.276	.074	2.27	.024
Conscientiousness	.144	4.89	<.001	.207	8.11	<.001	.117	3.73	<.001	.177	7.60	<.001	.314	11.51	<.001
Neuroticism	-.084	-1.84	.066	-.062	-1.57	.117	.010	0.21	.834	-.176	-4.90	<.001	-.107	-2.56	.011
Openness	-.046	-1.43	.152	-.032	-1.17	.242	-.027	-0.79	.432	.008	0.32	.746	.023	0.78	.435

*Note.* Gender was dummy-coded (0 = males, 1 = females).

It can be seen that there are several differences in the significant predictors between the regression models predicting the different NSI scales. However, the FEAR scale of the ANPS was the strongest (negative) predictor for the *Physiological*, *Safety and Security*, and *Esteem* scores of the NSI. For the NSI *Belonging* score, the strongest predictors were the PLAY (positively) and SADNESS scale of the ANPS (negatively) and the Agreeableness/Extraversion scales of the BFI (positively). For the NSI *Self-actualization* score, the strongest (positive) predictor was the Conscientiousness scale of the BFI. Overall, Conscientiousness out of the *Big Five* model was a *significant* positive predictor for each NSI scale score.

### 3.5 Comparison of the satisfaction with Maslow's needs, the importance of their satisfaction and the actual hierarchy of needs

**Figure 1:**
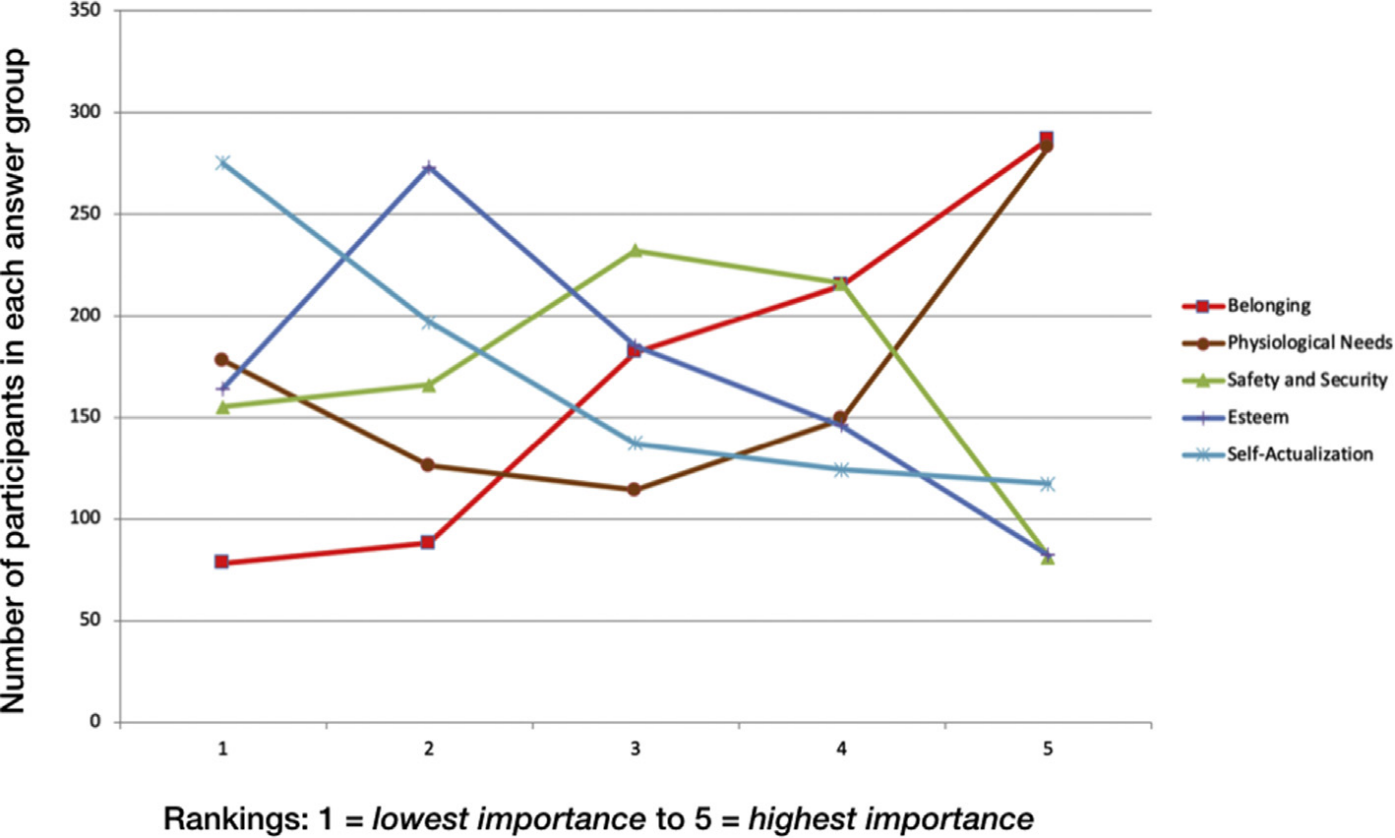
The figure depicts the number of participants in each ranking group with respect to the rated importance of Maslow's needs. To illustrate this with an example: Most participants provided *Self-actualization* with the lowest importance rating (1), whereas the need of *Belonging* was for most of the participants of such as importance that only few persons rated BB *Belonging* low).

When comparing the ranks of the mean scores of the NSI scales (assessing satisfaction with the five basic need categories) and the importance rating of the satisfaction of the basic need categories with the order for the proposed hierarchy of needs, there were several differences. Whereas *Self-actualization* is the final goal/need according to Maslow's proposed hierarchy, people rated *Belonging* as the most important basic need, but it ranked only 4^th^ in need satisfaction. *Self-actualization* was rated to be the least important basic need. Interestingly, *Physiological needs* were rated as the least satisfied basic need category but were rated as the second most important basic need category (see Table 5). For further illustrations, see Figure 1 below and Table S2 of the Supplementary Material. Beyond that we present in the supplement correlations between the BFI/ANPS and the NSI in different age groups (Tables S3 to S7). All data are available via the Maslow.sav (SPSS-file).

**Table 5:** Rank of the mean scores of the NSI scales (assessing satisfaction with the basic needs), the importance rating of the basic needs and the Maslow's hierarchy of needs.

Satisfaction (NSI scales)			Importance (rating of basic needs)			Maslow's Hierarchy of needs	
Rank	Scale	*M* (*SD*)	Rank	Scale	*M* (*SD*)	Rank	Scale
1	Esteem	4.34 (0.82)	1	Belonging	3.64 (1.29)	1	Self-actualization
2	Safety and Security	4.33 (0.77)	2	Physiological needs	3.27 (1.56)	2	Esteem
3	Self-actualization	4.32 (0.80)	3	Safety and Security	2.88 (1.24)	3	Belonging
4	Belonging	4.29 (0.80)	4	Esteem	2.66 (1.24)	4	Safety and Security
5	Physiological needs	4.07 (0.73)	5	Self-actualization	2.54 (1.42)	5	Physiological needs

## 4 Discussion

The present study was designed to investigate individual differences in Maslow's needs and their association with the prominent *Big Five* personality traits and the bottom-drivers, namely subcortically-anchored primary emotional systems as measured at the language level with the ANPS. From a theoretical point of view, this is important because personality traits are typically seen as relatively stable across the lifespan rather than shifting as a function of what prepotent personal needs have been satisfied (Borghuis et al., 2017; Dobewall and Aavik, 2016; Edmonds et al., 2008; Kawamoto and Endo, 2015; Specht et al., 2011; Wortman et al., 2012). However, individual personality traits could influence the extent to which people could satisfy the needs featured in Maslow's pyramid.

However, as Maslow expected, lower needs have to be satisfied before higher needs can emerge, and it is apparent in our data that FEAR, SADNESS, and the *Big Five*'s Neuroticism appear already at the *Physiological needs* level and persist through the "higher" levels of *Esteem* and *Self-actualization*.

Indeed, the trait of Neuroticism and its closely associated primary emotions, FEAR and SADNESS were robustly and negatively linked with all of Maslow's needs, suggesting that the negative emotionality highlighted at the *Safety and Security* level are likely to be a hindrance towards fulfillment of Maslow's needs in general. It was mentioned in the introduction above that phasic activation of SADNESS and FEAR is of importance for survival and helps ensure that our species and we as individuals are able to have a long life with the opportunity to achieve the highest levels in Maslow's need hierarchy. In contrast, chronic higher activation of these neural circuitries (to be expected by persons scoring high on these traits) will be a hindrance towards being satisfied with each of Maslow's needs.

In our data, positive associations could also be consistently seen between Extraversion, and its bottom up driver, the PLAY system, with Maslow's needs. Therefore, these positive personality traits seem to enable individuals to satisfy each of the needs featured in Maslow's pyramid.

On a theoretical note, the hierarchical regressions revealed that, after taking into account the ANPS primary emotional systems, only Conscientiousness from the *Big Five* model consistently (in terms of significance) added to the explained variance when predicting each of Maslow's needs. SEEKING was a highly significant predictor of Maslow's *Self-actualization.* PLAY was a highly significant predictor for Maslow's *Physiological*, *Safety* and *Belonging* needs, with Extraversion contributing more strongly for *Belonging*, *Esteem* and *Self-actualization.* To the extent that the *Big Five* scales reflect a higher-order (perhaps more cortical) representation of primary emotions, these results may reflect the idea that highest levels in Maslow's theory, namely, *Esteem* and *Self-actualization,* are more cognitively driven (although also robust negative associations with FEAR and SADNESS appeared), with the powerful SEEKING system perhaps bridging these levels, whereas the lower steps are under stronger emotional influence. In sum, it makes sense that primary emotional systems play a stronger role at the lower needs, whereas the *Big Five* traits come in at later needs with Conscientiousness performing the role of regulating primary emotions and putting the brakes on them when necessary to achieve a higher-level goal.

However, there is a nuance to this generalization: The SEEKING scale was the second most predictive variable after Conscientiousness in the *Self-actualization* regression equation. Both SEEKING and Conscientiousness reached their highest correlation with *Self-actualization* compared to all other Maslow needs. Perhaps Maslow's highest need represents exceptional passion (SEEKING) along with a strong capacity to focus and regulate competing interests (Conscientiousness). Those few who select the *Self-actualization* path to fulfillment may be combining one of the most potent emotions with the ability to moderate other emotions that might interfere with their aspirations. One could further speculate that Maslow's other four needs often represent alternate paths to fulfillment, that is, other "ways of being" that are satisfying different profiles of emotionally-based needs that constitute their personalities.

Of further interest, our study reveals that the participants in this study especially valued the *Need to belong* (and *Love need*), which emerged as the strongest need when participants were asked to rate which of Maslow's needs they view of particular importance in their lives. Here, a very different order appeared compared to Maslow's proposed hierarchy of needs, with people stating that, in their personal lives, *Safety*, *Self-esteem* and *Self-actualization* were the least important Maslow needs (see also the *Needs Ranking Chart*). In sum, participants don't agree with Maslow's view that *Self-actualization* is of highest importance in their lives (although its links to well-being have been documented in works such as by Kaufman (2018)). In this context, a recent work by Krems et al. (2017) points towards the fact that *Self-actualization* and Maslow's lower needs might be not as independent as originally proposed because, according to life history variables such as being in a relationship or not, having children or not might impact what lay persons understand as being self-actualized. According to the work by Krems et al. (2017), achieving status might just be “means to an end” to become self-actualized. However, in our study, we did not seek to understand what lay people understand by the concept of *Self-actualization*. We explained to our participants in the questionnaire how we understood *Self-actualization*, namely, “to grow as a person”. In this, we are very close to what Maslow understood by the concept and also as outlined in Table 4 presented in Krems' work (Krems et al., 2017). giving a list of exceptional persons such as Einstein or Gandhi as references of self-actualized persons. An additional note regarding the work underlying the Krems et al. (2017) paper is that the SEEKING system, which strongly predicted *Self-actualization,* was not included among their fundamental motives, likely because it was not a social motive, and Maslow's *Self-actualization* may not always be socially driven. Furthermore, they did not include Conscientiousness in their work, again likely because it was not a primary motive. A more Pankseppian affective neuroscience approach would see Conscientiousness as a regulator of primary motivators.

This said, we believe that our results, presented in the middle of Table 5, very much mirror the impact of our emotional heritage on everyday life. The impact of primary emotional systems on our desire to live in groups and to maintain stable relationships is secured by the activation of the SADNESS circuit when being separated from our loved ones, but also of the positive feeling accompanying activity of the neural circuit underlying CARE when we are loved and being taken care of. From Table 2, the *Need to belong* was positively correlated with CARE (r = .24, p < .001) and negatively correlated with SADNESS (r = -.33, p < .001). Of note, more than twenty years ago Pettijohn and Pettijohn (1996) observed very similar results in their survey. When students were asked which of Maslow's needs is of highest relevance in their lives, the majority (78%) said that “falling or staying in love with your ideal mate” comes in first.

We believe that our work has important implications. First, it shows that personality traits play an important role when trying to understand satisfaction of Maslow's needs. Second, our work underlines that different frameworks can be used to come to such an understanding. Here we investigated the widely used *Big Five* framework and a newer, more biologically grounded, personality framework based on AN theory. Finally, our work challenges the order of Maslow's pyramid.

The present study had several limitations. We administered self-report inventories to assess individual differences in the satisfaction levels (states) and personality dimensions (traits). Therefore, limits in self-monitoring ability, together with tendencies towards answering in a socially desirable fashion, might have biased the present results. This said, the replication of patterns between the *Big Five* traits and primary emotional systems, as presented in the supplementary material, shows that at least this part of the results seems to be reliable. Also the overall satisfactory psychometric properties speak for the quality of the scales assessing need satisfaction and personality traits (although an exception is the rather weak CFI of the NSI confirmatory factor analysis.^2^ All participants received feedback on their data in an online format as a compensation for their participation efforts. Providing wrong answers would lead to invalid feedback, and this is something the participants knew.

Aside from this, the present study is correlational in nature. Hence the suggested causal links between primary emotional systems and the *Big Five* have been derived against the background of affective neuroscience theory, but we cannot draw cause-and-effect conclusions. At best neuroscientific research endeavors might ultimately reveal the validity of our claims if combined with a longitudinal approach. A further limitation is the use of only one measure to assess satisfaction with Maslow's needs. Other measures have been published as well, and the use of these might have led to different results (e.g. Leidy, 1994). However, we believe that the replication of the already known links between Extraversion/Neuroticism and satisfaction with Maslow's needs in the present work speak against this limitation. Nevertheless, other self-report techniques have been proposed to locate a person at a given time point in Maslow's pyramid, and this might also impact on the results (Williams and Page, 1989).

Finally, the present administered questionnaires assessed Maslow's concepts as a state measure, whereas personality in the context of both *Big Five* and primary emotional systems have been operationalized as traits. Therefore, it could be expected that answering the *Need Satisfaction Inventory* might lead to more varying scores over time at the individual level compared to the *Big Five* and primary emotional traits, but this needs to be tested in future longitudinal research. Our results indicate that changes in need satisfaction over short periods of time might be small because: (1) the correlation between age and satisfaction with Maslow's needs were mostly small and (2) the research on life satisfaction has frequently indicated positive links between Extraversion and general life satisfaction (not linked to Maslow) and negative links between Neuroticism and general life satisfaction (e.g., a study with more than 40.000 participants by Lachmann et al., 2018).

With respect to life satisfaction, it has been pointed out that, in poorer countries, satisfaction with one's own financial situation might be more important than in richer countries (Oishi et al., 2009) and, therefore, in a future study, income level and other relevant SES parameters should be considered. Beyond that, only longitudinal works will ultimately shed light on the link between age and satisfaction with Maslow's needs. Krems et al. (2017) convincingly demonstrated that age clearly has an influence on the cognitive priority of different of Maslow's steps in the pyramid. But again, our studies differ in methods, and we could not see a dramatic age effect in our data.

In sum, to our knowledge, this work is the first to both link individual differences in satisfaction with Maslow's needs to the *Big Five* traits and their underlying emotional foundation in terms of Panksepp's primary emotional systems. The present work supports the idea that (chronic) energy from negative primary emotional systems (here assessed as personality traits) hinder the fulfillment of Maslow's needs, and brain energy related to positive primary emotional systems (in particular PLAY and SEEKING) might help persons achieve the different hierarchy levels of Maslow's needs. This said, activity in negative emotional brain circuits will be needed from time to time to ensure that people stay safe (activity of FEAR for fulfilment of *Safety needs* in the long run) or activity of SADNESS to reunite again with one's own close peer-group and family. Despite this, we are convinced that over activity of these systems for longer time periods will hinder the fulfillment of Maslow's needs.

Finally, conducting our research in different cultures might be worthwhile for a further understanding of what has been observed in our work. Future studies might also consider stronger the impact of socioeconomic status (Oishi et al., 2009).

Our research chose Panksepp's AN theory as an evolutionary framework, stemming from comparative mammalian brain research. Other approaches might be chosen in the future in the realm of co-varying personality traits, perhaps even needs of satisfaction, by taking a look at the niche diversity hypothesis (Lukaszewski et al. (2019)). Here it has been argued that socioecological complexity influences personality co-variation (Lukaszewski et al., 2017; Smaldino et al., 2019), such as the height of correlations of the *Big Five* or perhaps even across personality models.

In conclusion, this work demonstrates that Maslow's humanistic view on mankind might have been too positive. Most people strive for the *Need to belong/Love need* rather than *Self-actualization*, which in our work ranked last in importance. In our opinion this indicates how our evolutionary heritage still resonates in us and impacts on our lives.

## Declarations

### Author contribution statement

Christian Montag: Conceived and designed the experiments; Analyzed and interpreted the data; Contributed reagents, materials, analysis tools or data; Wrote the paper.

Cornelia Sindermann: Analyzed and interpreted the data; Wrote the paper.

David Lester, Kenneth Davis: Analyzed and interpreted the data; Contributed reagents, materials, analysis tools or data; Wrote the paper.

### Funding statement

This research did not receive any specific grant from funding agencies in the public, commercial, or not-for-profit sectors.

### Competing interest statement

The authors declare no conflict of interest. Dr. Montag has received (to Ulm University and earlier University of Bonn) grants from funding agencies such as the German Research Foundation (DFG). Moreover, Dr. Montag has performed grant reviews for several agencies; has edited journal sections and articles; has given academic lectures in clinical or scientific venues or companies; and has generated books or book chapters for publishers of mental health texts. For some of these activities he received royalties, but never from the gaming or social media industry. Finally, Dr. Montag mentions that he is part of a discussion circle (Digitalität und Verantwortung: https://about.fb.com/de/news/h/gespraechskreis-digitalitaet-und-verantwortung/) debating ethical questions linked to social media, digitalization and society/democracy at Facebook. In this context, he receives no salary for his activities. Finally, he mentions that he currently functions as independent scientist on the scientific advisory board of the Nymphenburg group. This activity is financially compensated.

### Additional information

No additional information is available for this paper.

## Appendix A Supplementary data

Maslow_Supplement Maslow_Supplement

Maslow.sav Maslow.sav

## Footnotes

1 For an overview of selected Pankseppian principles see Panksepp (2011); Davis and Montag (2019).

2 Note that it might be better to use an answer format with an odd number of answer options for the NSI.

## References

[bib1] Benson S.G., Dundis S.P. (2003). Understanding and motivating health care employees: integrating Maslow’s hierarchy of needs, training and technology. J. Nurs. Manag..

[bib2] Bogg T., Roberts B.W. (2004). Conscientiousness and health-related behaviors: a meta-analysis of the leading behavioral contributors to mortality. Psychol. Bull..

[bib3] Borghuis J., Denissen J.J., Oberski D., Sijtsma K., Meeus W.H., Branje S., Bleidorn W. (2017). Big Five personality stability, change, and codevelopment across adolescence and early adulthood. J. Pers. Soc. Psychol..

[bib4] Cesario J., Johnson D.J., Eisthen H.L. (2020). Your brain is not an onion with a tiny reptile inside. Curr. Dir. Psychol. Sci..

[bib5] Davis K.L., Montag C. (2018). A tribute to Jaak Panksepp (1943–2017). Personality Neuroscience.

[bib6] Davis K.L., Montag C. (2019). Selected principles of Pankseppian affective neuroscience. Front. Neurosci..

[bib7] Davis K.L., Panksepp J. (2011). The brain’s emotional foundations of human personality and the Affective Neuroscience Personality Scales. Neurosci. Biobehav. Rev..

[bib8] Davis K.L., Panksepp J. (2018). The Emotional Foundations of Personality: A Neurobiological and Evolutionary Approach.

[bib9] Davis K.L., Panksepp J., Normansell L. (2003). The affective neuroscience personality scales: normative data and implications. Neuro-psychoanalysis.

[bib10] DeCarvalho R.J., Maslow Abraham H. (1991). (1908-1970) an intellectual biography. Thought: Fordham Univ. Q..

[bib11] Dobewall H., Aavik T. (2016). Rank-order consistency and profile stability of self- and informant-reports of personal values in comparison to personality traits. J. Indiv. Differ..

[bib12] Edmonds G.W., Jackson J.J., Fayard J.V., Roberts B.W. (2008). Is character fate, or is there hope to change my personality yet?. Soc. Pers. Psychol. Compass.

[bib13] Gambrel P.A., Cianci R. (2003). Maslow’s hierarchy of needs: does it apply in a collectivist culture. J. Appl. Manag. Enterpren..

[bib14] Giacolini T., Ardizzone I., Davis K.L., Ferrara M., Picconi L., Terrinoni A., Sabatello U. (2017). Brain emotional systems: the Italian version of the ANPS-affective neuroscience personality scales 2.4 (reliability and validity). Clin. Neuropsychiatry.

[bib15] Hagerty M.R. (1999). Testing Maslow’s hierarchy of needs: national quality-of-life across time. Soc. Indicat. Res..

[bib16] Kaufman S.B. (2018). Self-actualizing people in the 21st century: integration with contemporary theory and research on personality and well-being. J. Humanist. Psychol..

[bib17] Kawamoto T., Endo T. (2015). Personality change in adolescence: results from a Japanese sample. J. Res. Pers..

[bib18] Kenrick D.T., Griskevicius V., Neuberg S.L., Schaller M. (2010). Renovating the pyramid of needs: contemporary extensions built upon ancient foundations. Perspect. Psychol. Sci..

[bib19] Kiel J.M. (1999). Reshaping Maslow’s hierarchy of needs to reflect today’s educational and managerial philosophies. J. Instr. Psychol..

[bib20] Knežević G., Lazarević L.B., Montag C., Davis K. (2020). Relations between lexical and biological perspectives on personality: new evidence based on HEXACO and affective neuroscience theory. J. Pers. Assess..

[bib21] Koltko-Rivera M.E. (2006). Rediscovering the later version of Maslow’s hierarchy of needs: self-transcendence and opportunities for theory, research, and unification. Rev. Gen. Psychol..

[bib22] Krems J.A., Kenrick D.T., Neel R. (2017). Individual perceptions of self-actualization: what functional motives are linked to fulfilling one’s full potential?. Pers. Soc. Psychol. Bull..

[bib23] Lachmann B., Sariyska R., Kannen C., Błaszkiewicz K., Trendafilov B., Andone I., Montag C. (2018). Contributing to overall life satisfaction: personality traits versus life satisfaction variables revisited — is replication impossible?. Behav. Sci..

[bib24] Leidy N.K. (1994). Operationalizing Maslow’s theory: development and testing of the basic need satisfaction inventory. Issues Ment. Health Nurs..

[bib25] Lester D. (1990). Maslow’s hierarchy of needs and personality. Pers. Indiv. Differ..

[bib26] Lester D., Hvezda J., Sullivan S., Plourde R. (1983). Maslow’s hierarchy of needs and psychological health. J. Gen. Psychol..

[bib27] Lukaszewski A.W., Gurven M., von Rueden C.R., Schmitt D.P. (2017). What explains personality covariation? A test of the socioecological complexity hypothesis. Social Psycholo. Personality Sci..

[bib28] Lukaszewski A., Gurven M., von Rueden C.R., Smaldino P. (2019). Toward Integration of the Niche Diversity Hypothesis With Other Explanations for Personality Covariation: reply to Međedović’s (2019) Commentary on Lukaszewski et al. (2017). Social Psycholo. Personality Sci..

[bib29] MacLean P.D. (1990). The Triune Brain in Evolution: Role in Paleocerebral Functions.

[bib30] Maslow A.H. (1943). A theory of human motivation. Psychol. Rev..

[bib31] Maslow A.H. (1969). The farther reaches of human nature. J. Transpers. Psychol..

[bib32] Maslow A.H. (1970). Motivation and Personality.

[bib33] Miles J., Shevlin M. (2001). Applying Regression & Correlation - A Guide for Students and Researchers.

[bib34] Montag C., Davis K.L. (2018). Affective neuroscience theory and personality: an update. Personality Neuroscience.

[bib35] Montag C., Davis K.L., Lazarevic L.B., Knezevic G. (2019). A Serbian version of the ANPS and its link to the five-factor model of personality. Open Psychol..

[bib36] Montag C., Elhai J.D. (2019). A new agenda for personality psychology in the digital age?. Pers. Indiv. Differ..

[bib37] Montag C., Panksepp J. (2017). Primary emotional systems and personality: an evolutionary perspective. Front. Psychol..

[bib38] Montag C., Widenhorn-Müller K., Panksepp J., Kiefer M. (2017). Individual differences in Affective Neuroscience Personality Scale (ANPS) primary emotional traits and depressive tendencies. Compr. Psychiatr..

[bib39] Noltemeyer A., Bush K., Patton J., Bergen D. (2012). The relationship among deficiency needs and growth needs: an empirical investigation of Maslow’s theory. Child. Youth Serv. Rev..

[bib40] Oishi S., Diener E., Lucas R.E., Suh E.M., Diener E. (2009). Cross-cultural variations in predictors of life satisfaction: perspectives from needs and values. Culture and Well-Being.

[bib41] Oleson M. (2004). Exploring the relationship between money attitudes and Maslow’s hierarchy of needs. Int. J. Consum. Stud..

[bib42] Panksepp J. (1998). Affective Neuroscience: the Foundations of Human and Animal Emotions.

[bib43] Panksepp J. (2011). Cross-species affective neuroscience decoding of the primal affective experiences of humans and related animals. PloS One.

[bib44] Panksepp J., Watt D. (2011). Why does depression hurt? Ancestral primary-process separation-distress (PANIC/GRIEF) and diminished brain reward (SEEKING) processes in the genesis of depressive affect. Psychiatr. Interpers. Biol. Process..

[bib45] Pettijohn T.F., Pettijohn T.F. (1996). Perceived happiness of college students measured by Maslow’s hierarchy of needs. Psychol. Rep..

[bib46] Rammstedt B., Danner D. (2016). Die Facettenstruktur des Big Five Inventory (BFI). Diagnostica.

[bib47] Reuter M., Panksepp J., Davis K.L., Montag C. (2017). ANPS: Affective Neuroscience Personality Scales - Deutsche Version.

[bib48] Saunders S., Munro D., Bore M. (1998). Maslow’s hierarchy of needs and its relationship with psychological health and materialism. South Pacific J. Psychol..

[bib49] Smaldino P.E., Lukaszewski A., von Rueden C., Gurven M. (2019). Niche diversity can explain cross-cultural differences in personality structure. Nature Human Behav.

[bib50] Specht J., Egloff B., Schmukle S.C. (2011). Stability and change of personality across the life course: the impact of age and major life events on mean-level and rank-order stability of the Big Five. J. Pers. Soc. Psychol..

[bib51] Taormina R.J., Gao J.H. (2013). Maslow and the motivation hierarchy: measuring satisfaction of the needs. Am. J. Psychol..

[bib52] Terracciano A., Löckenhoff C.E., Zonderman A.B., Ferrucci L., Costa P.T. (2008). Personality predictors of longevity: activity, emotional stability, and conscientiousness. Psychosom. Med..

[bib53] Williams D.E., Page M.M. (1989). A multi-dimensional measure of Maslow’s hierarchy of needs. J. Res. Pers..

[bib54] Wortman J., Lucas R.E., Donnellan M.B. (2012). Stability and change in the Big Five personality domains: evidence from a longitudinal study of Australians. Psychol. Aging.

