# Supplementary material for: Linking individual differences in satisfaction with each of Maslow's needs to the Big Five personality traits and Panksepp's primary emotional systems
Source: Heliyon. 2020 Jul 23;6(7):e04325. doi: 10.1016/j.heliyon.2020.e04325 (PMC7387820; doi:10.1016/j.heliyon.2020.e04325)
Supplement: Maslow_Supplement [file mmc1.docx]

Linking Individual Differences in Satisfaction With Each of Maslow’s Needs to the Big Five of Personality and Panksepp’s Primary Emotional Systems

**Supplementary Material**

Table S1

*Zero-order Pearson Correlations Between the ANPS and the BFI Scales*

|  |  | Extraversion | | | | | |  | Agreeableness | | | | | | |  | Conscientiousness | | | | | | | |  | Neuroticism | | | | | |  | Openness | | | | | |  |
| --- | --- | --- | --- | --- | --- | --- | --- | --- | --- | --- | --- | --- | --- | --- | --- | --- | --- | --- | --- | --- | --- | --- | --- | --- | --- | --- | --- | --- | --- | --- | --- | --- | --- | --- | --- | --- | --- | --- | --- |
|  |  | *r* | | | *p* | | |  | *r* | | | | *p* | | |  | *r* | | | | *p* | | | |  | *r* | | | *p* | | |  | *r* | | | *p* | | |  |
|  | SEEKING |  | .39 |  |  | < .001 |  |  |  | .16 |  |  |  | < .001 |  |  |  | .19 |  |  |  | < .001 |  |  |  |  | -.20 |  |  | < .001 |  |  |  | .46 |  |  | < .001 |  |  |
|  | FEAR |  | -.38 |  |  | < .001 |  |  |  | -.20 |  |  |  | < .001 |  |  |  | -.24 |  |  |  | < .001 |  |  |  |  | .74 |  |  | < .001 |  |  |  | -.05 |  |  | .181 |  |  |
|  | CARE |  | .22 |  |  | < .001 |  |  |  | .37 |  |  |  | < .001 |  |  |  | .06 |  |  |  | .076 |  |  |  |  | .07 |  |  | .048 |  |  |  | .26 |  |  | < .001 |  |  |
|  | ANGER |  | -.03 |  |  | .383 |  |  |  | -.50 |  |  |  | < .001 |  |  |  | -.22 |  |  |  | < .001 |  |  |  |  | .52 |  |  | < .001 |  |  |  | -.11 |  |  | < .001 |  |  |
|  | PLAY |  | .48 |  |  | < .001 |  |  |  | .29 |  |  |  | < .001 |  |  |  | .06 |  |  |  | .074 |  |  |  |  | -.34 |  |  | < .001 |  |  |  | .14 |  |  | < .001 |  |  |
|  | SADNESS |  | -.26 |  |  | < .001 |  |  |  | -.20 |  |  |  | < .001 |  |  |  | -.20 |  |  |  | < .001 |  |  |  |  | .68 |  |  | < .001 |  |  |  | -.01 |  |  | .850 |  |  |

Table S2

*Number of Answers With Respect to the Importance of Maslow’s Needs of All Participants*

|  |  | Ranking  1 | | | Ranking  2 | | | Ranking  3 | | | Ranking  4 | | | Ranking  5 | | |  |
| --- | --- | --- | --- | --- | --- | --- | --- | --- | --- | --- | --- | --- | --- | --- | --- | --- | --- |
|  | Belonging |  | 78 |  |  | 88 |  |  | 182 |  |  | 215 |  |  | 287 |  |  |
|  | Physiological needs |  | 178 |  |  | 126 |  |  | 114 |  |  | 149 |  |  | 283 |  |  |
|  | Safety and Security |  | 155 |  |  | 166 |  |  | 232 |  |  | 216 |  |  | 81 |  |  |
|  | Esteem |  | 164 |  |  | 273 |  |  | 185 |  |  | 146 |  |  | 82 |  |  |
|  | Self-Actualization |  | 275 |  |  | 197 |  |  | 137 |  |  | 124 |  |  | 117 |  |  |

*Note.* Hierarchy of Ranking; 1 = *Lowest Importance* and 5 = *Highest Importance.*

Table S3

*Correlations Between Maslow’s Physiological Needs (PN) and Dimensions of the BFI and ANPS*

|  |  | PN  1 | | | PN  2 | | | PN  3 | | | PN  4 | | | PN  (*n* = 850) | | |  |
| --- | --- | --- | --- | --- | --- | --- | --- | --- | --- | --- | --- | --- | --- | --- | --- | --- | --- |
|  | SEEKING |  | **0.397** |  |  | **0.273** |  |  | **0.264** |  |  | 0.183 |  |  | **0.279** |  |  |
|  | FEAR |  | **‑0.549** |  |  | **‑0.574** |  |  | **‑0.559** |  |  | **‑0.555** |  |  | **‑0.559** |  |  |
|  | CARE |  | -0.028 |  |  | 0.013 |  |  | 0.054 |  |  | -0.032 |  |  | 0.002 |  |  |
|  | ANGER |  | -0.190 |  |  | **-0.338** |  |  | **-0.281** |  |  | **-0.352** |  |  | **-0.290** |  |  |
|  | PLAY |  | **0.432** |  |  | **0.511** |  |  | **0.358** |  |  | **0.284** |  |  | **0.396** |  |  |
|  | SADNESS |  | **-0.565** |  |  | **-0.531** |  |  | **-0.517** |  |  | **-0.519** |  |  | **-0.533** |  |  |
|  | Extraversion |  | **0.380** |  |  | **0.320** |  |  | **0.203** |  |  | **0.280** |  |  | **0.296** |  |  |
|  | Agreeableness |  | **0.208** |  |  | **0.323** |  |  | 0.108 |  |  | 0.165 |  |  | **0.201** |  |  |
|  | Conscientiousness |  | **0.368** |  |  | 0.164 |  |  | **0.316** |  |  | **0.321** |  |  | **0.292** |  |  |
|  | Neuroticism |  | **-0.528** |  |  | **-0.546** |  |  | **-0.472** |  |  | **-0.500** |  |  | **-0.512** |  |  |
|  | Openness |  | -0.014 |  |  | 0.023 |  |  | 0.081 |  |  | 0.062 |  |  | 0.038 |  |  |

*Note.* Pearson correlations >= .20 are printed in black bold letters; correlations >= .40 are printed in red bold letters; age-group 1: <= 22 (*n* = 172), age-group 2: > 22 til <= 35 (*n* = 233), age-group 3: > 35 til <= 50 (*n* = 267), age-group 4: > 50 (*n* = 178).

Table S4

*Correlations Between Maslow’s Safety and Security Needs (SS) and Dimensions of the BFI and ANPS*

|  |  | SS  1 | | | SS  2 | | | SS  3 | | | SS  4 | | | SS  5  (*n* = 850) | | |  |
| --- | --- | --- | --- | --- | --- | --- | --- | --- | --- | --- | --- | --- | --- | --- | --- | --- | --- |
|  | SEEKING |  | **0.390** |  |  | **0.308** |  |  | 0.175 |  |  | 0.191 |  |  | **0.266** |  |  |
|  | FEAR |  | **‑0.631** |  |  | **‑0.687** |  |  | **‑0.670** |  |  | **‑0.648** |  |  | **‑0.659** |  |  |
|  | CARE |  | -0.017 |  |  | 0.059 |  |  | -0.032 |  |  | -0.067 |  |  | -0.014 |  |  |
|  | ANGER |  | **-0.280** |  |  | **-0.273** |  |  | **-0.322** |  |  | **-0.322** |  |  | **-0.299** |  |  |
|  | PLAY |  | **0.423** |  |  | **0.470** |  |  | **0.424** |  |  | **0.384** |  |  | **0.425** |  |  |
|  | SADNESS |  | **-0.600** |  |  | **-0.605** |  |  | **-0.629** |  |  | **-0.572** |  |  | **-0.602** |  |  |
|  | Extraversion |  | **0.370** |  |  | **0.316** |  |  | **0.268** |  |  | **0.235** |  |  | **0.297** |  |  |
|  | Agreeableness |  | **0.211** |  |  | **0.370** |  |  | 0.176 |  |  | 0.186 |  |  | **0.236** |  |  |
|  | Conscientiousness |  | **0.460** |  |  | 0.193 |  |  | **0.398** |  |  | **0.348** |  |  | **0.350** |  |  |
|  | Neuroticism |  | **-0.592** |  |  | **-0.599** |  |  | **-0.556** |  |  | **-0.582** |  |  | **-0.582** |  |  |
|  | Openness |  | 0.027 |  |  | 0.049 |  |  | 0.025 |  |  | 0.016 |  |  | 0.029 |  |  |

*Note.* Pearson correlations >= .20 are printed in black bold letters; correlations >= .40 are printed in red bold letters; age-group 1: <= 22 (*n* = 172), age-group 2: > 22 til <= 35 (*n* = 233), age-group 3: > 35 til <= 50 (*n* = 267), age-group 4: > 50 (*n* = 178).

Table S5

*Correlations Between Maslow’s Belonging Need (B) and Dimensions of the BFI and ANPS*

|  |  | B  1 | | | B  2 | | | B  3 | | | B  4 | | | B  5  (*n* = 850) | | |  |
| --- | --- | --- | --- | --- | --- | --- | --- | --- | --- | --- | --- | --- | --- | --- | --- | --- | --- |
|  | SEEKING |  | **0.294** |  |  | **0.256** |  |  | 0.171 |  |  | 0.040 |  |  | 0.190 |  |  |
|  | FEAR |  | **‑0.344** |  |  | **‑0.423** |  |  | **‑0.272** |  |  | **‑0.304** |  |  | **‑0.336** |  |  |
|  | CARE |  | **0.315** |  |  | **0.235** |  |  | 0.193 |  |  | **0.251** |  |  | **0.249** |  |  |
|  | ANGER |  | -0.128 |  |  | -0.173 |  |  | -0.158 |  |  | **-0.358** |  |  | **-0.204** |  |  |
|  | PLAY |  | **0.512** |  |  | **0.523** |  |  | **0.345** |  |  | **0.325** |  |  | **0.426** |  |  |
|  | SADNESS |  | **-0.291** |  |  | **-0.430** |  |  | **-0.289** |  |  | **-0.330** |  |  | **-0.335** |  |  |
|  | Extraversion |  | **0.512** |  |  | **0.503** |  |  | **0.305** |  |  | **0.254** |  |  | **0.394** |  |  |
|  | Agreeableness |  | **0.399** |  |  | **0.357** |  |  | **0.288** |  |  | **0.367** |  |  | **0.353** |  |  |
|  | Conscientiousness |  | **0.212** |  |  | 0.140 |  |  | **0.273** |  |  | **0.354** |  |  | **0.245** |  |  |
|  | Neuroticism |  | **-0.293** |  |  | **-0.408** |  |  | **-0.235** |  |  | **-0.403** |  |  | **-0.335** |  |  |
|  | Openness |  | -0.018 |  |  | 0.143 |  |  | 0.134 |  |  | 0.058 |  |  | 0.079 |  |  |

*Note.* Pearson correlations >= .20 are printed in black bold letters; correlations >= .40 are printed in red bold letters; age-group 1: <= 22 (*n* = 172), age-group 2: > 22 til <= 35 (*n* = 233), age-group 3: > 35 til <= 50 (*n* = 267), age-group 4: > 50 (*n* = 178).

Table S6

*Correlations between Maslow’s esteem need (E) and dimensions of the BFI and ANPS*

|  |  | E  1 | | | E  2 | | | E  3 | | | E  4 | | | E  5  (*n* = 850) | | |  |
| --- | --- | --- | --- | --- | --- | --- | --- | --- | --- | --- | --- | --- | --- | --- | --- | --- | --- |
|  | SEEKING |  | **0.448** |  |  | **0.392** |  |  | **0.337** |  |  | **0.297** |  |  | **0.369** |  |  |
|  | FEAR |  | **‑0.586** |  |  | **‑0.704** |  |  | **‑0.658** |  |  | **‑0.638** |  |  | **‑0.647** |  |  |
|  | CARE |  | 0.090 |  |  | 0.139 |  |  | 0.057 |  |  | 0.058 |  |  | 0.086 |  |  |
|  | ANGER |  | -0.170 |  |  | **-0.268** |  |  | **-0.362** |  |  | **-0.381** |  |  | **-0.295** |  |  |
|  | PLAY |  | **0.458** |  |  | **0.521** |  |  | **0.490** |  |  | **0.333** |  |  | **0.451** |  |  |
|  | SADNESS |  | **-0.521** |  |  | **-0.587** |  |  | **-0.613** |  |  | **-0.567** |  |  | **-0.572** |  |  |
|  | Extraversion |  | **0.540** |  |  | **0.574** |  |  | **0.499** |  |  | **0.418** |  |  | **0.508** |  |  |
|  | Agreeableness |  | 0.198 |  |  | **0.398** |  |  | 0.196 |  |  | **0.235** |  |  | **0.257** |  |  |
|  | Conscientiousness |  | **0.465** |  |  | **0.312** |  |  | **0.370** |  |  | **0.434** |  |  | **0.395** |  |  |
|  | Neuroticism |  | **-0.528** |  |  | **-0.665** |  |  | **-0.609** |  |  | **-0.660** |  |  | **-0.616** |  |  |
|  | Openness |  | -0.002 |  |  | 0.166 |  |  | 0.166 |  |  | 0.195 |  |  | 0.131 |  |  |

*Note.* Pearson correlations >= .20 are printed in black bold letters; correlations >= .40 are printed in red bold letters; age-group 1: <= 22 (*n* = 172), age-group 2: > 22 til <= 35 (*n* = 233), age-group 3: > 35 til <= 50 (*n* = 267), age-group 4: > 50 (*n* = 178).

Table S7

*Correlations between Maslow’s self-actualization need (SA) and dimensions of the BFI and ANPS*

|  |  | SA  1 | | | SA  2 | | | SA  3 | | | SA  4 | | | SA  5  (*n* = 850) | | |  |
| --- | --- | --- | --- | --- | --- | --- | --- | --- | --- | --- | --- | --- | --- | --- | --- | --- | --- |
|  | SEEKING |  | **0.587** |  |  | **0.427** |  |  | **0.327** |  |  | **0.353** |  |  | **0.424** |  |  |
|  | FEAR |  | **‑0.383** |  |  | **‑0.474** |  |  | **‑0.430** |  |  | **‑0.535** |  |  | **‑0.456** |  |  |
|  | CARE |  | 0.194 |  |  | **0.251** |  |  | **0.229** |  |  | 0.130 |  |  | **0.201** |  |  |
|  | ANGER |  | -0.140 |  |  | -0.184 |  |  | **-0.264** |  |  | **-0.403** |  |  | **-0.248** |  |  |
|  | PLAY |  | **0.373** |  |  | **0.473** |  |  | **0.362** |  |  | **0.327** |  |  | **0.384** |  |  |
|  | SADNESS |  | **-0.365** |  |  | **-0.411** |  |  | **-0.353** |  |  | **-0.526** |  |  | **-0.414** |  |  |
|  | Extraversion |  | **0.473** |  |  | **0.571** |  |  | **0.398** |  |  | **0.375** |  |  | **0.454** |  |  |
|  | Agreeableness |  | **0.209** |  |  | **0.345** |  |  | **0.263** |  |  | **0.340** |  |  | **0.289** |  |  |
|  | Conscientiousness |  | **0.578** |  |  | **0.392** |  |  | **0.452** |  |  | **0.466** |  |  | **0.472** |  |  |
|  | Neuroticism |  | **-0.369** |  |  | **-0.459** |  |  | **-0.390** |  |  | **-0.638** |  |  | **-0.464** |  |  |
|  | Openness |  | 0.092 |  |  | **0.222** |  |  | **0.202** |  |  | **0.279** |  |  | 0.199 |  |  |

*Note.* Pearson correlations >= .20 are printed in black bold letters; correlations >= .40 are printed in red bold letters; age-group 1: <= 22 (*n* = 172), age-group 2: > 22 til <= 35 (*n* = 233), age-group 3: > 35 til <= 50 (*n* = 267), age-group 4: > 50 (*n* = 178).
