# Supplementary material for: Linking individual differences in satisfaction with each of Maslow's needs to the Big Five personality traits and Panksepp's primary emotional systems
Source: Heliyon. 2020 Jul 23;6(7):e04325. doi: 10.1016/j.heliyon.2020.e04325 (PMC7387820; doi:10.1016/j.heliyon.2020.e04325)
Supplement: Appendix [file mmc3.docx]

# Appendix

# German Version of the Need Satisfaction Scale

Table A1

*German Version of the Need Satisfaction Scale*

| Item No. | Item |
| --- | --- |
| 1. | Ich habe abends niemals Probleme einzuschlafen. |
| 2. | Ich denke, dass die Welt heutzutage ein ziemlich sicherer Platz ist. |
| 3. | Ich weiß, dass mich meine Familie immer unterstützen und auf meiner Seite sein wird; egal was auch passiert. |
| 4. | Ich bin die meiste Zeit unzufrieden mit mir. |
| 5. | Ich habe eine gute Vorstellung davon, was ich im Leben machen möchte. |
| 6. | Mein Einkommen reicht aus, um meine Bedürfnisse zu befriedigen. |
| 7. | Ich würde nachts nicht alleine durch meine Nachbarschaft gehen. |
| 8. | Ich habe eine bedeutsame Liebesbeziehung zu einem/einer Anderen. |
| 9. | Ich fühle mich von meinem Umfeld respektiert. |
| 10. | Mein Leben hat Bedeutung. |
| 11. | Ich bekomme ausreichend Erholung. |
| 12. | Mein Ängstlichkeitslevel ist hoch. |
| 13. | Ich fühle mich ohne Wurzeln. |
| 14. | Ich fürchte nur selten, dass meine Handlungen dazu führen, dass meine Freunde eine geringe Meinung von mir haben. |
| 15. | Ich bin mir über meine Ziele im Leben nicht sicher. |
| 16. | Ich habe ein zufriedenstellendes Sexualleben. |
| 17. | Ich fühle, dass die Menge an Geld, die ich zur Verfügung habe und verdiene, mir Sicherheit bringt. |
| 18. | Ich habe eine Gruppe von Freunden, mit denen ich Dinge unternehme. |
| 19. | Ich kann auf eigenen Beinen stehen. |
| 20. | Ich fühle, dass ich mein Potential ausschöpfe. |
| 21. | Generell ist meine Gesundheit gut. |
| 22. | Ich fühle mich sicher und geborgen. |
| 23. | Ich fühle mich ein wenig sozial isoliert. |
| 24. | Ich fühle mich in meinem gegenwärtigen Betätigungsfeld selbstsicher. |
| 25. | Ich strebe nach Reife. |
| 26. | Im Winter ist es mir immer zu kalt. |
| 27. | Ich habe Angst davor, nachts alleine in meinem Haus / meiner Wohnung zu sein. |
| 28. | Ich habe einige wenige enge Freunde, auf die ich mich verlassen kann. |
| 29. | Ich würde mich als eine selbstbewusste Person beschreiben. |
| 30. | Ich finde meine Arbeit herausfordernd. |
| 31. | Ich esse genug, um meine physiologischen Bedürfnisse zu befriedigen. |
| 32. | Mein Leben ist geordnet und gut strukturiert. |
| 33. | Ich fühle mich meinen Verwandten nahe. |
| 34. | Ich habe mir den Respekt von anderen verdient. |
| 35. | Ich weiß was ich kann und was ich nicht kann. |
| 36. | Ich komme ausreichend dazu, Sport zu machen. |
| 37. | Ich kann mich darauf verlassen, dass mir andere helfen, wenn ich Hilfe benötige. |
| 38. | Ich bin an meiner ethnischen Herkunft interessiert und fühle mich mit meiner ethnischen Gruppe verwandt. |
| 39. | Ich verbringe nicht viel Zeit damit, darüber zu grübeln, was andere von mir denken. |
| 40. | Ich fühle, dass ich das Beste gebe was mir möglich ist. |
| 41. | Üblicherweise macht mir irgendein Körperteil Beschwerden. |
| 42. | Ich bin oftmals über meine körperliche Gesundheit besorgt. |
| 43. | Ich bin religiös und bin Mitglied einer Glaubensgemeinschaft. |
| 44. | Ich fühle, dass ich eine wertvolle Person bin. |
| 45. | Ich fühle, dass ich als Person reife. |
| 46. | Die Sommer sind mir immer zu heiß, um mich wohl zu fühlen. |
| 47. | Mein Leben verläuft im positiven Sinne routiniert. |
| 48. | Es ist mir möglich, zumindest einem nahestehenden und vertrauten Freund meine innersten Gedanken und Gefühle anzuvertrauen. |
| 49. | In Gruppen fühle ich für gewöhnlich, dass meine Meinung den Meinungen von anderen unterlegen ist. |
| 50. | Meine akademischen Erfolge sind vor dem Hintergrund meiner Fähigkeiten angemessen. |

*Note.* Response Scale: (-3) *stimme überhaupt nicht zu*, (-2), (-1), (+1), (+2), (+3) *stimme sehr zu.*
